# 

**Published:** 1878-10

**Authors:** 


### Books and Pamphlets Received.

---

Barnes on Diseases of Women. Henry C. Lea. T. H. Butler.

Antagonism of Medicines. By J. Milner Fothergill. Henry C. Lea. T. H. Butler.

Quantitive Analysis. By Alexander Classen and Edgar F. Smith. Henry C. Lea. T. H. Butler.

Proceedings of the Medical Society of the County of Kings.

Conspectus of the different forms of Phthisis. By Roswell Park, A. M., M. D.

College of Physicians and Surgeons. New York. Seventy-first Annual Catalogue.

Transactions of the Twenty-fifth Annual Meeting of the Medical Society of the State of North Carolina.

Battey's Operation. Three fatal cases with some remarks upon the indications for the operation. By George J. Engelmann

Ninth Annual Announcement of the Woman's Hospital Medical College, Chicago.

Prospectus of the New York College of Pharmacy.

Henry Gibbons, Sr., M. D., on Feticide.

Removal of Naso-Pharyngeal Polypus by temporary depression of both upper Jaws. By L McLane Tiffany.

Report on the Health of Children in the Oneida Community. By T. R. Noyes, M. D.

The Pith of the dried Corn Stalk as a Uterine tent. By W. T. Goldsmith, M. D.

Medico-Legal Evidence Relating to the Detection of Human Blood. By Joseph Jones, M. D.

Berberidaceæ, the Botanical description, commercial history, medical properties and pharmaceutical preparations, by C. G. and J. U. Lloyd. Cincinnati. 16 pages.

A Treatise on Milk, and Henri Nestle's Milk Food, for the earliest period of Infancy, and in later years, by H. Lebert, Medical Privy Councillor and Professor. Page 38.

Cholecystotomy for the removal of Gall Stones in Dropsy of the Gall, Bladder, by J. Marion Sims, M. D. London: T. Richards, 37 Great Queen St. 1878. pp. 20.

Upon the Treatment of Strumous Disease, by what may be called the Solfatara method, by Horatio R. Storer, M. D. Newport, R. I.

Annual Reports of the Supervising Surgeon General of the Marine Hospital Service of the United States, for the fiscal years, 1876 and 1877. John M. Woodworth, M. D.

075

### THE BUFFALO

### MEDICAL AND SURGICAL

MEDICAL AND SURGICAL JOURNAL.

PUBLISHED MONTHLY,—Containing Original Articles, Reports of Medical Societies and Hospitals, Editorial, Reviews, Correspondence, Army News, etc., etc; including the usual variety of Medical Periodical Publications. Specimen copies sent on application to Buffalo Medical and Surgical Journal, J. F. MINER, M. D., 978 Main Street, BUFFALO, N. Y.

TERMS OF SUBSCRIPTION, $3 00 PER YEAR, IN ADVANCE,—All communications for publication should be sent before the fifteenth (15) of each month, or they will of necessity lie over until the next number. No change can be made in advertisements after the twenty-fifth.

Correspondence and original articles are solicited. Publication is no evidence that the views of the author are endorsed by the Journal.

